# Spatiotemporal dynamics of PDGFRβ expression in pericytes and glial scar formation in penetrating brain injuries in adults

**DOI:** 10.1111/nan.12539

**Published:** 2019-04-02

**Authors:** C. Reeves, A. Pradim‐Jardim, S. M. Sisodiya, M. Thom, J. Y. W. Liu

**Affiliations:** ^1^ Department of Clinical and Experimental Epilepsy UCL Queen Square Institute of Neurology Queen Square London; ^2^ Department of Neuropathology UCL Queen Square Institute of Neurology Queen Square London; ^3^ Department of Neurology and Neurosurgery Universidade Federal de Sao Paulo UNIFESP Sao Paulo/SP Brazil; ^4^ Chalfont Centre for Epilepsy Chesham Lane Chalfont St Peter Bucks SL9 0RJ UK; ^5^ School of life Sciences University of Westminster London W1W 6UW UK

**Keywords:** Brain injury, glial scar, pericytes, progenitors

## Abstract

**Aims:**

Understanding the spatiotemporal dynamics of reactive cell types following brain injury is important for future therapeutic interventions. We have previously used penetrating cortical injuries following intracranial recordings as a brain repair model to study scar‐forming nestin‐expressing cells. We now explore the relationship between nestin‐expressing cells, PDGFRβ^+^ pericytes and Olig2^+^ glia, including their proliferation and functional maturation.

**Methods:**

In 32 cases, ranging from 3 to 461 days post injury (dpi), immunohistochemistry for PDGFRβ, nestin, GFAP, Olig2, MCM2, Aquaporin 4 (Aq4), Glutamine Synthetase (GS) and Connexin 43 (Cx43) was quantified for cell densities, labelling index (LI) and cellular co‐expression at the injury site compared to control regions.

**Results:**

PDGFRβ labelling highlighted both pericytes and multipolar parenchymal cells. PDGFRβ LI and PDGFRβ^+^/MCM2^+^ cells significantly increased in injury Zones at 10–13 dpi with migration of pericytes away from vessels with increased co‐localization of PDGRFβ with nestin compared to control regions (*P* < 0.005). Olig2^+^/MCM2^+^ cell populations peaked at 13 dpi with significantly higher cell densities at injury sites than in control regions (*P* < 0.01) and decreasing with dpi (*P* < 0.05). Cx43 LI was reduced in acute injuries but increased with dpi (*P* < 0.05) showing significant cellular co‐localization with nestin and GFAP (*P* < 0.005 and *P* < 0.0001) but not PDGFRβ.

**Conclusions:**

These findings indicate that PDGFRβ^+^ and Olig2^+^ cells contribute to the proliferative fraction following penetrating brain injuries, with evidence of pericyte migration. Dynamic changes in Cx43 in glial cell types with dpi suggest functional alterations during temporal stages of brain repair.

## Introduction

There is a fine balance in repair processes following brain injury between the beneficial effects that limit tissue damage and restore homeostasis and the long‐term detrimental effects that can follow glial scar formation, such as impediment to axonal regeneration and increased risk of seizures 1, 2. Understanding which endogenous progenitor cell types contribute to tissue reorganization after brain injury and the mechanisms that regulate their differentiation and functional maturation are essential for any therapeutic interventions targeting this process.

Most studies of the spatiotemporal progression of glial and vascular alterations have been carried out using experimental animal models of brain injury 2, 3, 4 and are dependent on the type of model used and, together with inter‐species differences, are not always translatable to human brain injury. Studies investigating brain repair in human tissues have largely been dependent on postmortem material with its inherent limitations. We have previously overcome this by utilizing surgical specimens with penetrating cortical injuries following intracranial recordings (ICR) carried out for the preoperative investigation of epilepsy 5, 6. As the time interval between initial electrode placement and subsequent tissue resection is precisely known (but variable between cases) and the injury is localized, this provides a unique spectrum from acute injuries to chronic scars, spanning from 3 days to over 1‐year‐old injuries, to study spatiotemporal processes in human brain repair. Our previous studies identified that, in addition to microglia, nestin‐expressing cells represent a highly proliferative cell type at the acute injury site, often elongated with a close relationship to vessels and remaining in chronic scars; a proportion of these nestin‐expressing cells were immunopositive for GFAP 5. In experimental injury models, functionally distinct subtypes of astroglia are recognized, regulating inflammatory responses and repair, including specific ‘scar‐forming astrocytes’ with elongated processes 3, 7. The source of proliferating and scar‐forming glia in injuries is not fully established. Progenitor cell types in the adult brain, including oligodendroglial progenitor cells (OPCs), NG‐2 cells as well as resident parenchymal nestin‐expressing glia with proliferative capacity 8, are candidates. A further possibility, particularly in view of the observed intimate relationship of new capillaries and glia in the repair process, is that nestin‐expressing cells are recruited from progenitors in the perivascular niche, including pericytes with capacity for neuroectodermal differentiation 9.

Pericytes form an essential part of the neurovascular unit, responsible for regulating the capillary blood flow through controlling capillary structure and diameter, promoting the development and maintenance of the blood‐brain barrier and controlling the proliferation, migration and stabilization of endothelial cells 10, 11, 12, 13. Pericytes may be divided into different subtypes based on their function, morphology, location and expression of immunohistochemical markers including platelet‐derived growth factor receptor beta (PDGFRβ), alpha smooth muscle actin (α‐SMA), chondroitin sulphate proteoglycan (NG2), regulator of G protein signalling (RGS‐5) and the potassium channel Kir6.1 14, 15, 16; they have also been reported to express nestin in neonatal and adult rat brain cultures 17, 18. PDGFRβ expressing pericytes have been proposed to be an important source of endogenous progenitor cells 9, 13, 19, 20, capable of enhanced proliferation following injury in experimental models 10, 18, 21. However, a recent study showed that Tbx18^+^ pericytes did not behave as stem cells, including following brain injury and repair 22, highlighting an ongoing controversy in this area.

As reactive glial progenitors proliferate and differentiate following injury to form the chronic scar, the temporal expression of functional markers, for example those involved in gliotransmission, could be relevant to any local cellular dysfunction as well as epileptogenesis 23. Cx43 is the main gap junction protein in astrocytes, establishing homeostatic cell communication 24. Cx43 hemichannels also mediate ‘gliotransmission’ of bioactive molecules 25, 26 and astroglial connexins and Aquaporin 4 are implicated in spreading depolarization that can follow brain injury 27, 28. Glutamate synthetase, while integral to normal glial glutamate metabolism and excitatory neuronal transmission, is deficient in glial progenitor types 29.

Using our series of ICR injury cases we hypothesized that (i) OPC and PDGFRβ expressing pericytes contribute to the proliferative cell types following focal injury, (ii) their spatiotemporal distribution, morphology and dynamics are closely related to GFAP‐negative nestin‐expressing cells and (iii) relative differences in markers of functional maturation between reactive glial populations over the time course of brain repair could be observed.

## Materials and methods

The project has ethical approval and written informed consent was obtained from all patients.

### Case selection

Surgical brain specimens from 32 patients, with pharmacoresistant focal epilepsy, who had placement of subdural and/or depth electrodes during preoperative intracranial EEG recordings at various periods prior to resective surgery between 2002 and 2015, were included in the study. All samples were received through the Epilepsy Society Brain and Tissue Bank at the Department of Neuropathology, UCL Queen Square Institute of Neurology. The lesion age in each case was recorded as days post injury (dpi; interval between the placement of the electrodes and tissue resection) which ranged from 3 to 461 days. Cases were also arbitrarily grouped into four temporal stages of cellular injury and repair for qualitative analyses and to enable comparison with our previous studies 5: acute (3–9 dpi; *n* = 6), subacute (10–13 dpi; *n* = 7), intermediate (28–70 dpi; *n* = 5) and chronic lesions (107–461 dpi; *n* = 14). The clinical details of the cases are presented in Table 1; 19 cases were male and the mean age at surgery was 36 years (range, 18–60 years). The injury types involved the superficial cortex including the sub‐pial layer (following subdural grid placements; *n* = 14) or were needle‐like track penetrating injuries (from depth electrode insertion; *n* = 19) (Table 1). Most tissue resections were from the frontal (*n* = 17) and temporal (*n* = 12) lobes. In 13 cases, no distinct pathology was identified in the resection (nonlesional); the remaining cases showed focal cortical dysplasia (FCD) type IIB (*n* = 10), FCD IIID (*n* = 1), hippocampal sclerosis (*n* = 3), low‐grade epilepsy‐associated tumour (LEAT) (*n* = 4) and a focal scar from traumatic brain injury (*n* = 1). In all cases except one (case E22), the ICR lesion was *not* located in the same area as the main pathology.

**Table 1:** Clinical details of cases and marker combinations for immunohistochemical studies. Under Pathology, nonlesional refers to cases with no remarkable pathology

STAGE	Days post injury	Case	Sex	Age at surgery (years)	Type of injury	Pathology	Lobe	Immunohistochemical & Immunofluorescent studies				
								Nestin & PD or GFAP (IF)	Mcm2 & Olig2 (IF)	Mcm2 & PD (DAB/VIP)	Aq4, GS or Cx43 (DAB)	Cx43 & Nestin, PD or GFAP (IF)
ACUTE	3	E1	M	49	SUP	LEAT (DNT)	Fr	✓	✓		✓	✓
	6	E2^b^	F	23	SUP	NONLESIONAL	Fr	✓	✓		✓	
	8	E3^b^	F	18	SUP	FCDIIB	Fr‐P	✓	✓		✓	✓
	8	E4^b^	F	30	SUP^+^ DEEP	NONLESIONAL	T	✓		✓	✓	✓
	8	E5	M	32	SUP	FCD IIB	P	✓		✓		
	9	E6	M	39	SUP	NONLESIONAL	Fr	✓	✓		✓	
SUBACUTE	10	E7	F	23	SUP	NONLESIONAL	T	✓	✓	✓		
	10	E8	F	24	SUP	FCD IIB	T	✓	✓	✓		
	10	E9	F	34	DEEP	FCDIIB	Fr			✓	✓	
	11	E10^b^	M	40	SUP	FCD IIB	Fr	✓	✓	✓	✓	✓
	12	E11	M	27	SUP	OLD TBI	Fr	✓				
	12	E12	M	30	SUP	NONLESIONAL	Fr	✓	✓		✓	
	13	E13	M	29	SUP	FCDIIB	Fr	✓	✓	✓	✓	✓
INTERMEDIAETE	28	E14	F	59	DEEP	NONLESIONAL	Fr	✓	✓			
	30	E15	F	22	DEEP	HS	T	✓	✓		✓	
	30	E16	F	31	SUP	FCDIIB	Fr				✓	✓
	52	E17	M	25	DEEP	NONLESIONAL	O	✓	✓	✓		
	70	E18	M	35	DEEP	HS	T	✓	✓	✓	✓	✓
CHRONIC	107	E19	F	44	SUP	NONLESIONAL	T	✓				
	175	E20	F	60	DEEP	LEAT (MENINGIOANGIOMATOSIS)	T	✓				
	186	E21^b^	F	23	DEEP	NONLESIONAL	Fr	✓				
	209	E22	M	52	DEEP	FCDIIB^a^	Fr	✓	✓	✓		
	209	E23^b^	M	47	DEEP	NONLESIONAL	Fr	✓			✓	
	209	E24	M	25	DEEP	FCD IIB	T			✓		
	211	E25	M	32	DEEP	NONLESIONAL	Fr	✓				
	232	E26	F	53	DEEP	LEAT (DNT)	Fr	✓	✓			
	264	E27	M	21	DEEP	FCDIIB	Fr	✓			✓	✓
	301	E28	M	34	DEEP	NONLESIONAL	T	✓	✓	✓		
	329	E29	M	49	DEEP	HS	T	✓	✓			
	385	E30	M	55	DEEP	NONLESIONAL	T	✓			✓	
	417	E31	M	87	DEEP	FCDIIID	Fr	✓			✓	✓
	461	E32	M	49	DEEP	LEAT	T	✓			✓	
*In vitro* studies	n/a	EC1	M	49	n/a	HS	T	EdU A and Nestin immunocytochemistry				
	n/a	EC2	F	20	n/a	FCDIIB	Fr					

Aq4, aquaporin 4; Cx43, connexin 43; DAB, diaminobenzidine; DNT, Dysembryoplastic neuroepithelial tumour; FCDIIB, Frontal Cortical Dysplasia type IIB; F, female; Fr, frontal lobe; GS, glutamine synthetase, HS, hippocampal sclerosis; IF, immunofluorescent studies; M, male; Mcm2, mini chromosomal maintenance 2; O, occipital lobe; P, parietal lobe; PD, platelet‐derived growth factor receptor beta (PDGFRβ); SMA, smooth muscle actin; T, temporal lobe.

a The ICR injury was within the lesion in this case. In one case (E4) injuries in both the superficial and deep cortex were analysed separately.

b PDGFRβ and SMA immunofluorescence was performed on these cases.

### Immunohistochemical and double‐label immunofluorescence studies

Five‐micron thick serial sections were cut from each block bearing the injury; one was stained with haematoxylin and eosin as a reference for the location of the ICR lesion as well as to delineate the anatomical boundaries of three Zones: lesion core and margins (Zones 1 and 2) and control region. Further serial sections were used in single and double‐labelled chromogenic and immunofluorescence studies. The protocols and methods used were as published in previous studies 5, 8 (Supplementary methods). Antibodies used in single or double‐labelled studies included glial/glial progenitor markers, nestin, GFAP, Olig2; pericyte markers PDGFRβ 13, 21 and SMA; proliferative cell marker, MCM2 and functional glial markers, Glutamine synthetase (GS), Aquaporin4 (Aq4) and astrocyte gap junction protein Connexin 43 (Cx43) (Supplementary Table). It was not possible to include every case in all immunohistochemical and immunofluorescence studies as ICR lesions were small and some were ‘cut‐out’ in serial sections. However, for each study, a comparable number of cases from each of the four time periods were included (detailed in Table 1).

### Quantitative analyses

The distribution and morphology of single and double‐labelled cell types were visualized using Nikon brightfield microscope, Zeiss Axio Imager Z2 motorized fluorescence microscope and LSM700 confocal microscope (Zeiss, Oberkochen, Germany) and compared between differing Zones, age of lesions and the temporal stages of brain repair.

#### PDGFRβ, nestin, GFAP and Cx43 double‐labelled immunofluorescent sections

**Figure 1:**
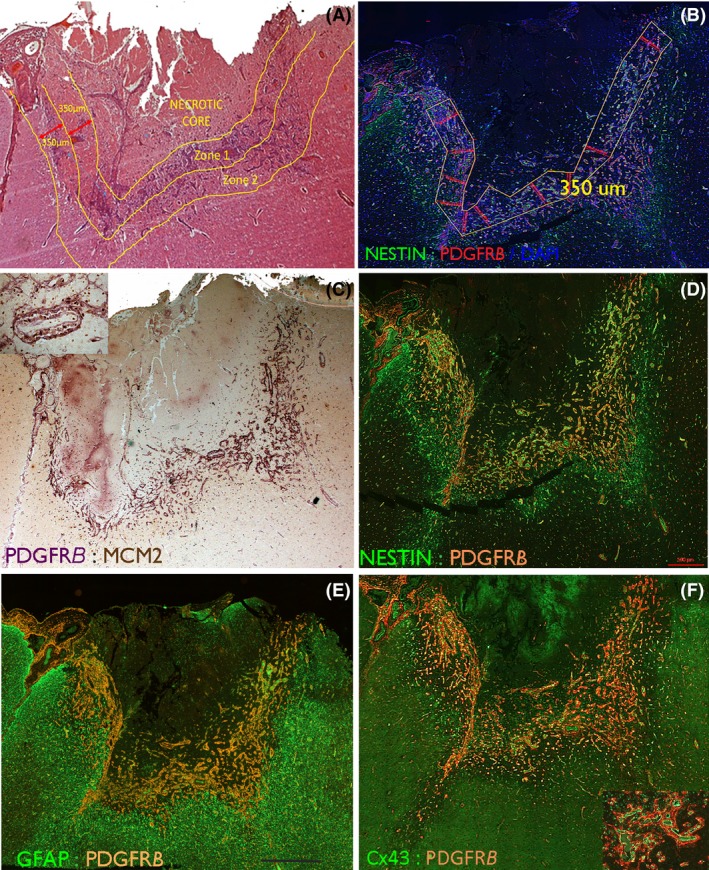
Intracranial recording (ICR) injury at low power. (**A**) A subacute lesion (13 days post injury) involving the cortical surface which represents one of the larger foci of injury in the series. H&E section highlighting the main regions used for qualitative evaluation and delineation of the Zones for study. The Necrotic core (Zone 0) contains mainly nonviable material and macrophages. Zone 1 is the rim of viable material surrounding the core and contains reactive cells and vessels and Zone 2 surrounds Zone 1. (**B**) The same case showing the superimposed measured Zone 1 with a width of 350 microns which was tiled for image analysis. (**C**) PDGFRβ chromogenic stained section (purple) with MCM2 labelling of nuclei (brown, visible in inset) in the same case to highlight the labelling in Zone 1 of vessels and in (**D**). With immunofluorescent labelling for PDGFRβ with nestin highlighting capillaries and reactive glial cells in Zone 1. (**E**) GFAP with PDGFRβ shows the relative compartmentalization of labelling at low power with the majority of GFAP positivity external to the PDGFRβ. (**F**) Cx43 with PDGFR β at low power and shown in the inset at higher magnification, with a close relationship between PDGFRB pericytes surrounding Cx43‐positive endothelium. Higher magnifications for C are shown in Figure 4, D in Figure 2, E in Figure 3 and F in Figure 5. Bar is 700 microns.

Each immunolabelled slide was tiled using a 20× objective and regions of interest were drawn as described in previous studies 5 using Zen imaging software (Carl Zeiss Microscopy, Jena, Germany). Zone 1 refers to the margin of the ICR lesion, the internal border formed by the medial edge of viable tissue to a radial depth of 350 μm, forming a circumferential region surrounding the injury site (Figure 1 **A,B**). The precise shapes and size of Zones drawn varied between cases, according to the contours of the ICR injury. Zone 2 represented tissue of equal radial depth marginal to Zone 1 (qualitative evaluation only) and Zone 3 was a control region of 2000 μm^2^ located as far away from the ICR injury site as possible within normal‐appearing tissue in the same tissue section. Care was taken to select a comparable region of control white matter or grey matter according to the ICR lesion site. Fluorescent signal thresholds were set for each fluorochrome using the Zen imaging software and were kept consistent across cases. For each set of double‐labelling studies, the labelling index (LI), rather than cell numbers, was measured since most markers labelled abundant cell processes in the brain parenchyma. In addition, the relative area of co‐localization (RAC; the area of co‐labelling/total area of Zone 1 or Zone 3) and co‐localization coefficients were calculated including Pearson's and Mander's coefficients as measures of the degree of co‐localization, where 1 indicates perfect overlap of signal, −1 indicates a negative correlation and 0 indicates random overlap 30. A proportion of cases were re‐tested between two observers with good reproducibly (intra‐class correlation coefficient 0.995). For Olig2/MCM2 sections, the same procedure for acquisition of the Zones was performed, but the number of double‐labelled cells per area was manually counted.

#### MCM2/PDGFRβ double‐labelled chromogenic sections

Slides were scanned with a Leica SCN400F digital slide scanner (Leica Microsystems, Wetzlar, Germany) at 40× magnification and immunolabelling in the same regions of interests as described above was analysed using the automated image analysis software, Definiens Tissue Studio software 3.6 (Definiens AG, Munich, Germany). Double‐labelled cells were automatically counted as MCM2^+^ brown nuclear labelling (MCM2) when surrounded by PDGFRβ^+^ cytoplasmic purple labelling and the data were expressed as the number of cells per area.

#### GS, Aq4 and Cx43 single labelling

Serial images were manually captured at 40× magnification from three regions of interest: Zone 0 (injury core), Zone 1 (organizing edge of the lesion) and Zone 3. Immunolabelling was thresholded using ImageProPlus (v6.3; MediaCybernetics, Bethesda, MD, USA) and the LI for each area calculated.

Statistical analyses were performed using SPSS for windows version 21 (IBM corporation, version 21) and significance between groups was taken at *P < *0.05. Tests included Kruskal–Wallis non‐parametric tests with *post‐hoc* correction for multiple comparisons and Wilcoxon signed rank to compare cases between different Zones. Spearman's correlation and regression analysis were used to explore the relationships between labelling and dpi.

## *In vitro* scratch assay

To investigate the response to injury after 24 h, a scratch assay was performed on cells cultured from a gram of freshly resected brain tissue of two surgical patients with focal epilepsy (Cases EC1, EC2; Table 1) and the proportion, morphology and spatial distribution of nestin‐expressing cells incorporating EdU A were quantified (see supplemental methods).

## Results

### Pericytes and glial cell reactions in ICR injuries

**Figure 2:**
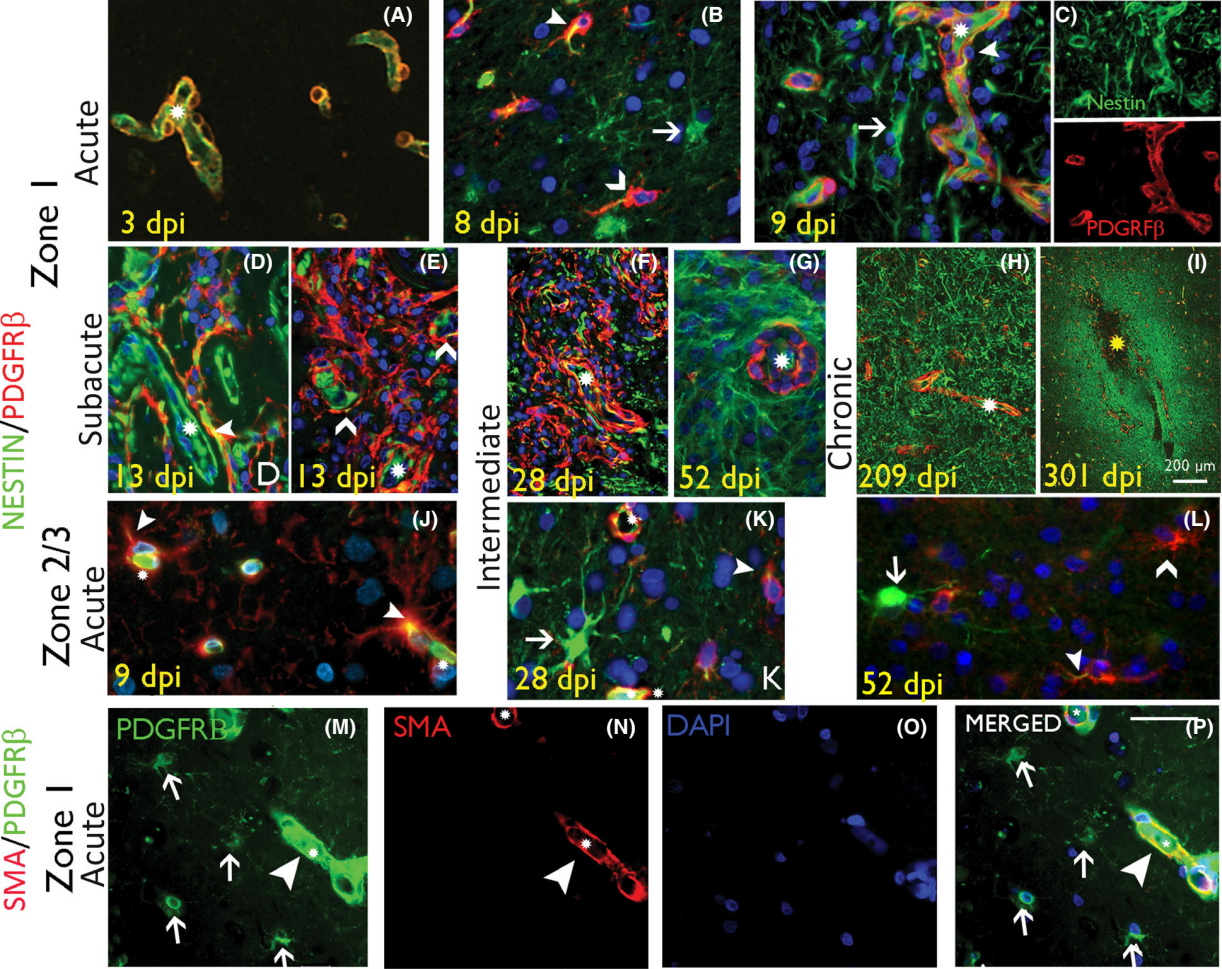
Pericytes and nestin‐expressing reactive glial cell types in intracranial recording (ICR) injuries of different stages. (**A–C**) Nestin/PDGFRβ in acute injuries in Zone 1. (**A**) At 3 days post injury (dpi) nestin labelling was relatively confined to vessel endothelium and PDGFRβ in pericytes, forming an incomplete layer around the endothelium. (**B‐C**) At 8 to 9 dpi, increased nestin expression in perivascular, reactive and bipolar stromal cells (arrows) with increased PDGFRβ cells around small vessels (chevrons), some with focal nestin co‐expression (arrowhead; asterisks indicate capillary lumen); In (**C**) the nestin and PDGFRβ are also shown as single channels. (**D, E**) Nestin/PDGFRβ in subacute injuries in Zone 1. (**D**) At this stage nestin expression is noted in elongated processes and reactive, bipolar and multipolar cells between capillaries and co‐expression with PDGFRβ is observed (arrowhead; split channel images shown in Figure S2). (**E**) ‘Lace‐like’ proliferations of PDGFRβ^+^ cells lifting away from the vasculature (asterisk) is noted (chevrons) (split channels shown in Figure S2). (**F**) Nestin/PDGFRβ in intermediate‐age injuries in Zone 1. By this stage more prominent increase in the number and networks of PDGFRβ^+^ cells in Zone 1 is noted, not associated with vessels (asterisk) and with some focal nestin co‐expression (split channels shown in Figure S2); these cells declined in number with age and, in (**G**) shown at 52 dpi, PDGFRβ expression is more limited to vasculature (asterisk). (**H** and **I**) Nestin/PDGFRβ in chronic injuries in Zone 1 at 209 and 301 dpi respectively, shows a residual increase in nestin^+^ fibrous processes, demarcating the scar site whereas PDGFRβ expression is mainly perivascular. (**J**) Nestin/PDGFRβ in acute injuries in Zone 3 in acute (8 dpi) and intermediate phases (**K** 28 dpi and **L** 52 dpi) shows multipolar cells that are nestin^+^ (arrow), PDGFRβ^+^ (chevron) or show double labelling (arrowhead) (asterisks indicate capillary) (**J**–**L** : shown at higher magnification in Figure S2C’). (**M–P**) SMA/PDGFRβ double labelling in Zone 1 confirms co‐localization in pericytic cells surrounding capillaries (arrowheads) but not in small multipolar PDGBRβ^+^ cells (arrows). In all figures, asterisks denote capillary vascular channels, arrowheads denote double‐labelled cells and arrows and chevrons denote single labelled cells as indicated. Scale bar shown in P is equivalent to 500 microns in A–G, 1000 and 20 microns in J to P.

**Figure 3:**
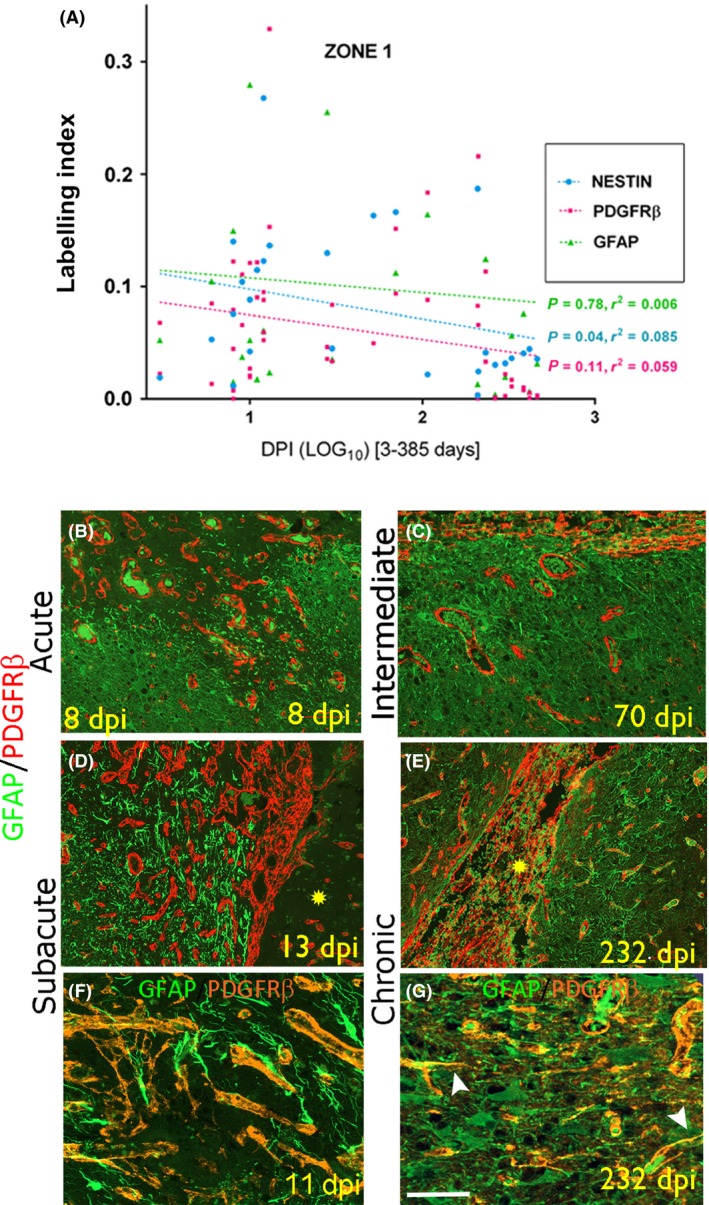
Pericytes and GFAP‐expressing reactive glial cell types in intracranial recording (ICR) injuries of different stages. (A) Scatter graphs of labelling index of nestin, PDGFRβ and GFAP plotted against dpi (expressed as log10); only nestin showed a significant regression with the age of the lesion. GFAP/PDGFRβ in Zone 1: **B** (acute), **D** (subacute), **C** (intermediate) and **E** (chronic) stages. The necrotic core of the penetrating injury is highlighted with yellow asterisk. Increased numbers of GFAP ^+^ fibres and PDGFRβ expressing cells are intermingled but form distinct populations at low magnification. Shown at higher magnification in **F** (subacute) and **G** (chronic, same case as E) shows approximation with intermingled and overlapping networks of GFAP and PDGFRβ processes in Zone 1 with apparent co‐localization in single processes (arrowheads Figure G) although orthogonal views were not available. Scale bar shown in G equivalent to 100 microns in B to E and 50 microns in F and G.

**Figure 4:**
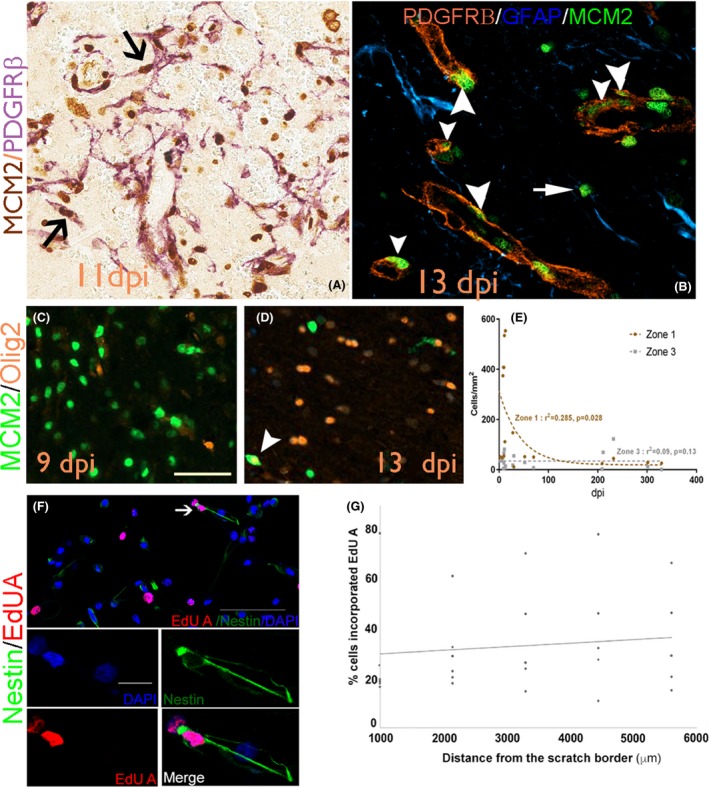
(**A**) MCM2/PDGFRβ at 11 days post injury with the lace‐like networks of cells, some of which show double labelling (arrows); (**B**) Double labelling for MCM2 with PDGFRβ at 13 dpi confirms double‐labelled cells in perivascular location (arrowheads) in addition to MCM2/PDGFRβ^‐^ cells (arrow). (**C**) MCM2/Olig2 at 9 dpi and (**D**) at 13 dpi showing double‐labelled cell (arrowhead). (**E**) Scatter graph of MCM2/Olig2 double‐labelled cell density (/mm^2^) with dpi showing a significant declined in Zone 1 (*P* = 0.028). (**F**) Confocal images showing nestin‐expressing cells grown from surgical temporal cortex of patients with focal epilepsy underwent cell division and incorporated EdU A, 24 hours after mechanical scratch injury. (**G**) Quantification showed that more dividing cells were noted further away from the scratch. Scale bar in C is 60 microns in A‐D; scale bar in F is 100 μm and scale bar in (blue channel) is 20 μm.

*Qualitative analysis:* Nestin/PDGFRβ: In acute ICR lesions, multipolar nestin^+^ cells and nestin^+^ cells with elongated processes became prominent between capillaries in Zones 1 and 2 (Figure 2 **A–C**); in Zone 3, nestin labelling was noted mainly in capillaries (Figure 2 **J**). In subacute to intermediate ICR lesions, there was greater prominence of nestin^+^ cells between new capillaries in both Zones 1 and 2 (Figure 1 **D**, 2 **D–G**). In chronic ICR lesions, dense meshworks of nestin^+^ fibres persisted in Zone 1 around vessels at the scar site (Figure 2 **H,I**) as previously described 5. PDGFRβ in acute lesions showed focal expression in pericyte‐like cells around capillaries in Zones 1 and 2 at 3 dpi (Figure 2 **A**). By 8–9 dpi, increased numbers of PDGFRβ^+^ bipolar cells and processes were present around capillaries (Figure 2 **B,C**) and by 13 dpi forming lace‐like meshworks extending away from capillaries, becoming more prominent in intermediate‐aged lesions (Figures 1 **C–F**, 2 **D–F**). In addition, in Zones 2 and 3, smaller multipolar PDGFRβ^+^ glial‐like cells not associated with capillaries were identified (Figure 2 **J–M**). In chronic lesions and oldest scars, PDGFRβ expression at the scar site became mainly limited to perivascular pericytes (Figure 2 **H**). Co‐localization of nestin with PDGFRβ was noted around acute, subacute and intermediate lesions in a proportion of the elongated cells in proximity to capillaries in Zone 1 (Figure 2 **C,D**). In addition, the lace‐like processes extending between capillaries in Zone 1 (Figure 2 **D–F**) and multipolar cells in Zones 2 and 3 also showed focal co‐expression (Figure 2 **J–L**). The small multipolar PDGFRβ cells, however, were not SMA positive, with PDGFRβ/SMA co‐expression restricted to pericyte‐like cells (Figure 2 **M–P**). GFAP/PDGFRβ: At low power, GFAP^+^ cells and processes formed a clear band of labelling at the boundary of Zones 1 and 2 in subacute injuries, mainly peripheral to an inner region of PDGFRβ^+^ cells and new capillaries, forming distinct compartments at low power (Figure 1 **E**). At higher magnification in Zone 1 of acute to chronic lesions, although many GFAP^+^ reactive processes appeared separate from PDGFRβ (Figure 3 **B–E**), intermingling and overlapping of processes with focal co‐localization was noted (Figure 3 **F,G**). GFAP and PDGFRβ^+^ cells also appeared relatively distinct in Zone 3. MCM2: PDGFRβ^+^/MCM2^+^ pericytes were prominent in subacute lesions in the immediate vicinity of capillaries and also in the elongated cells extending out from and between small capillaries (Figures 1 **C**, 4 **A**). Small Olig2^+^ cells with nuclear MCM2 co‐expression (Figure 4 **C,D**), indicating replicative potential, were also prominent in the ICR lesion site compared to normal cortex. The overall morphology of Olig2^+^ cells did not alter with dpi, although labelling was restricted to the nucleus.

Quantitative analysis: Nestin, PDGFRβ, GFAP. Nestin and PDGFRβ LI were significantly higher in Zone 1 (injury region) than Zone 3 (control region) in all cases (*P* < 0.001), but GFAP LI was not different (*P* = 0.526) (Table 2). There was a significant regression of nestin expression with dpi in Zone 1 (*P* < 0.04; Figure 3 **A**) but not in Zone 3 (Figure S3A), nor for GFAP or PDGFRβ. There was a significant correlation between nestin and PDGFRβ LI for all cases in Zone 1 (*P* < 0.001) but not with GFAP. There was a significantly greater relative area of co‐localization of nestin/PDGFRβ (*P* = 0.003) and GFAP/PDGFRβ in Zone 1 than Zone 3 (*P* = 0.02) in all ICR lesions over the time stages (Table 2; Figure S3B) and the Pearson correlation coefficient for co‐localization for nestin/PDGFRβ was significantly higher than for GFAP/PDGFRβ (*P* = 0.047, Wilcoxon test). There was a trend for increased co‐localization between GFAP/PDGFRβ and decreased nestin/PDGFRβ with dpi, but this was not significant.

**Table 2:** Quantitative findings from intracranial recording (ICR) lesions at stages of repair and control region

Cellular marker(s)	ICR injury region (time periods of injury)				Control region	
	Acute Mean (SD)	Subacute Mean (SD)	Int. Mean (SD)	Chronic Mean (SD)	Zone 3 Mean (SD)	Sig. (Z1 or Z2 to Z3)^a^
NESTIN LI ZONE 1	0.067 (0.049) *N* = 6	0.128 (0.075) *N* = 6	0.125 (0.056) *N* = 4	0.045 (0.048) *N* = 11	0.007 (0.008)	*P* < 0.000
GFAP LI ZONE 1	0.062 (0.054) *N* = 6	0.140 (0.163) *N* = 6	0.134 (0.111) *N* = 3	0.08 (0.115) *N* = 10	0.12 (0.09)	n/s
PDGFRβ LI ZONE 1	0.072 (0.042) *N* = 6	0.130 (0.103) *N* = 6	0.093 (0.053) *N* = 3	0.057 (0.082) *N* = 10	0.019 (0.01)	*P* < 0.000
NESTIN/PDGFRβ RAC ZONE 1	0.915 (1.6) *N* = 5	0.159 (0.11) *N* = 5	0.09 (0.14) *N* = 4	0.107 (0.107) *N* = 5	0.019 (0.03)	*P* = 0.003
GFAP/PDGFRβ RAC ZONE 1	0.34 (0.42) *N* = 4	0.37 (0.57) *N* = 5	0.75 (0.49) *N* = 3	1.01 (0.88) *N* = 7	0.35 (0.49)	*P* = 0.02
MCM2/PDGFRβ (Co‐labelled cells/mm^2^) Z1	236 (65) *N* = 2	604 (131) *N* = 5	379 (411) *N* = 2	433 (322) *N* = 3	147 (162)	*P* ≤ 0.005
MCM2/Olig2 (Co‐labelled cells/mm^2^) Z1	157 (187) *N* = 3	344 (212) *N* = 6	64 (58) *N* = 4	30 (9.1) *N* = 5	34 (33) *N* = 18	*P* < 0.01
Cx43 ZONE 0 Cx43 ZONE 1	0.12 (0.1) 0.29 (0.17) *N* = 6	0.12 (0.13) 0.25 (0.14) *N* = 4	0.12 (0.06) 0.46 (0.046) *N* = 3	0.41 (0.39) 0.39 (0.21) *N* = 4	0.32 (0.17) *N* = 17	*P* ≤ 0.005 n/s
Aq4 ZONE 0 Aq4 ZONE 1	0.14 (0.11) 0.23 (0.05) *N* = 6	0.08 (0.07) 0.34 (0.17) *N* = 4	0.17 (0.19) 0.3 (0.15) *N* = 3	0.06 (0.1) 0.26 (0.06) *N* = 5	0.30 (0.09) *N* = 18	*P* ≤ 0.001 n/s
GS ZONE 0 GS ZONE 1	0.09 (0.07) 0.29 (0.15) *N* = 5	0.2 (0.21) 0.22 (0.15) *N* = 4	0.18 (0.16) 0.26 (0.15) *N* = 3	0.1 (0.14) 0.24 (0.02) *N* = 4	0.23 (0.11) *N* = 16	n/s
Cx43/NESTIN (RAC) ZONE 1	1.78 (1.5) *N* = 3	5.1 (3.3) *N* = 3	7.13 *N* = 1	20.4 (2.9) *N* = 2	1.05 (1.3) *N* = 9	*P* < 0.05
Cx43/GFAP (RAC) ZONE 1	3.7 (4.5) *N* = 3	12.4 (9.1) *N* = 3	b	49.4 (13.1) *N* = 2	18.7 (11.6) *N* = 8	n/s
Cx43/PDGFRβ (RAC) ZONE 1	5.3 (3.9) *N* = 3	8.1 (8.3) *N* = 3	7.8 *N* = 1	12.3 (7.5) *N* = 2	3.8 (3.1) *N* = 9	n/s

Statistical differences are shown for the four time periods for each marker(s) and also compared to the control region (for all lesions of all ages). Cx43, connexin43; Z1, Z2, Zones 1 and 2 ICR injury site; Z3, Zone 3 control site (see text for details of delineation of each Zone on different stained sections); INT, intermediate; *N*, number of cases; LI, Labelling Index; RAC, relative area of co‐localization (see method section for detail); Sig., statistical significance.

Kruskal Wallis test for differences between four time periods in Zone 1.

a Wilcoxon Signed Ranks Test for differences between Zone 1 or 2 (as indicated) from Zone 3 for all cases/time periods).

b Analysis not conducted.

MCM2: There was a significant increase in MCM2/PDGFRβ^+^ cell density in Zone 1 compared to Zone 3 (*P* < 0.005); mean densities peaked in the subacute lesions in Zone 1 (Table 2) but there was no significant correlation of MCM2/PDGFRβ^+^ cells with dpi in either Zone. The density of Olig2/MCM2^+^ cells was significantly higher in Zone 1 than Zone 3 (*P* = 0.006); their number peaked at 13 dpi in subacute lesions with densities ten times higher than in normal regions (Table 2). By 28 dpi, the density of MCM2/Olig2^+^ cells had markedly decreased and continued to fall in chronic lesions to levels comparable with normal regions, with a significant regression shown with dpi in Zone 1 (Figure 4 **E**; exponential *P* = 0.028). *In vitro* scratch assay: The responses of proliferative cell populations 24 h following injury were further explored *in vitro,* since tissue samples with 1 dpi lesions were rarely available. Thirty‐three percent of the cells cultured from resected samples from two patients with focal epilepsy incorporated EdU A 24 h after the scratch. Ten percent of proliferative EdU A incorporated cells also expressed nestin (Figure 4 **F**). The majority of EdU A/nestin^+^ cells showed unipolar or bipolar morphology and did not appear to be apoptotic. These cells were found up to 7000 μm away from the induced lesion site. More EdU A labelled cells seem to be located further away from the scratch border than at the lesion site (Figure 4 **G**).

### Functional glial maturation in ICR injuries

**Figure 5:**
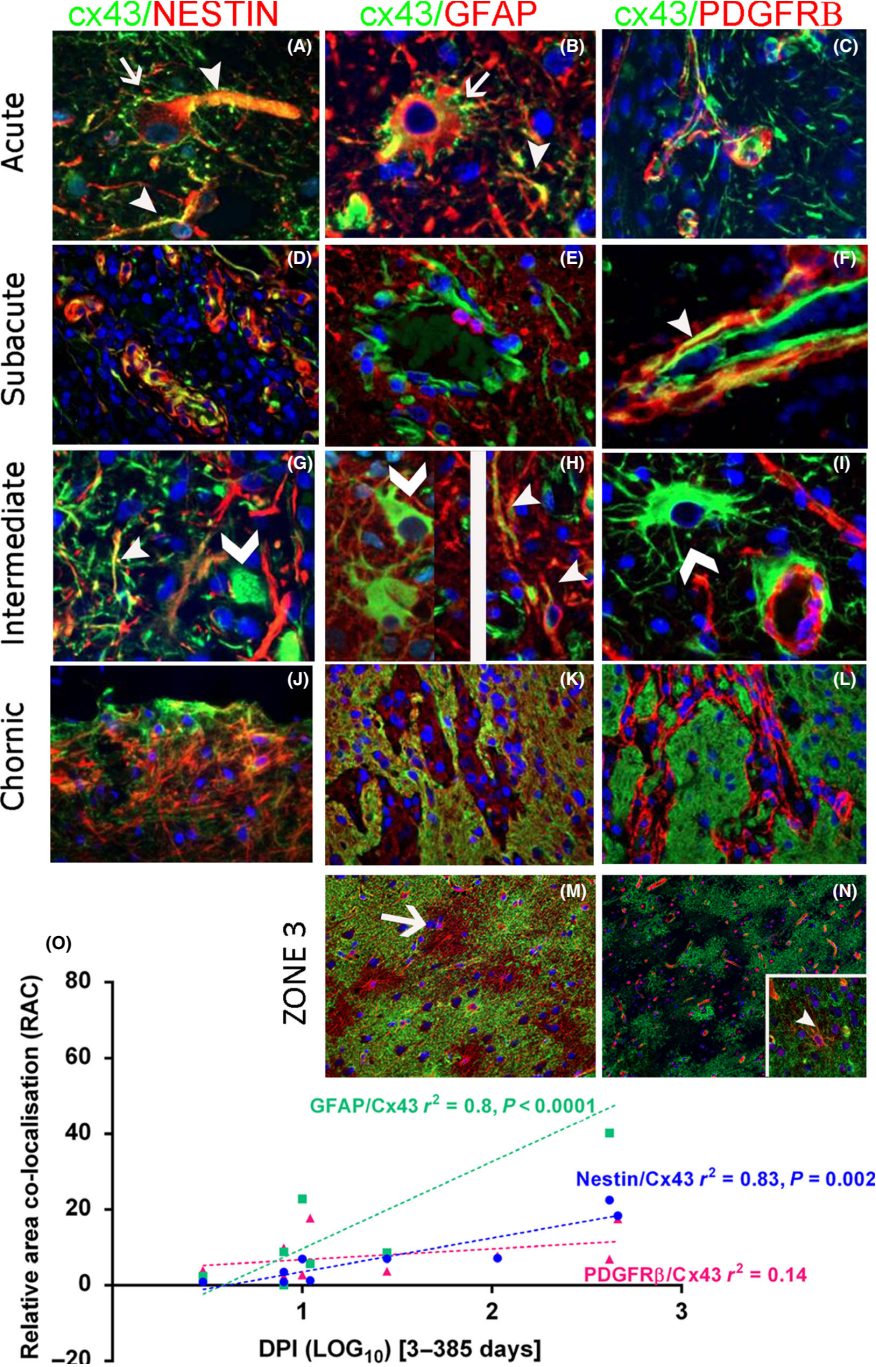
Cx43 labelling in glial cell types in intracranial recording lesions (ICR). Staining patterns in different stages of repair all in Zone 1 (margin of the ICR lesion): acute 8 days post injury (dpi) (**A**–**C**), subacute 11 dpi (**D**–**F**), intermediate 30 dpi (**G–I**) and chronic 417 dpi (**J–L**). Nestin/Cx43: (**A**) In acute lesions, peripheral Cx43 labelling was noted around large reactive nestin‐expressing cells (arrow) with focal co‐localization in processes (arrow head) (Split channel shown in Figure S3F). (**D**) In subacute lesions more prominent focal co‐localization in endothelium and perivascular cells was noted; this was still evident in elongated process in intermediate‐age ICR lesions (arrowhead) although some large Cx43‐positive cells were negative for nestin (chevron) (**G**). (**J**) In chronic lesions co‐expression was noted in a proportion (but not all) nestin cells at the scar site. GFAP/Cx43: (**B**) In acute lesions, marginal expression of Cx43 was noted around large reactive GFAP ^+^ cells (arrow) as well as co‐localization in some processes (arrowhead) (Split channel shown in Figure S3E). (**E**) In subacute and (**H**) intermediate lesions, co‐labelling of processes was noted (arrowheads) although many large Cx43‐positive reactive appearing cells (chevron) were negative for GFAP. (**K**) In chronic lesions, a more general overlap between the meshwork of glia fibres in the scar and Cx34 was observed. PDGFRβ/Cx43: (**C**) In acute lesions there were many Cx43 processes that were not PDGFRβ‐positive. (**F**) In subacute lesions some co‐localization was noted in pericyte‐like cells around capillaries (arrowhead; shown in split channels in Figure S3D). (**I**) In intermediate lesions, lack of co‐labelling is appreciated between large reactive Cx43^+^ cells (chevron) cells and foot processes around capillary pericytes and in (**L**) this was also appreciated in chronic lesions. Control Zone 3: (**M**) Extensive labelling is noted with Cx43 in the cortex however a proportion of GFAP astrocytic domains are devoid of Cx43 labelling. (**N**) Patchy or nonconfluent labelling with Cx43 is noted and separate from the PDGFRβ in capillaries; (inset) occasional multipolar cell shows co‐localization. (**O**) Linear regression analysis for the relationship between co‐localization of the three markers (GFAP, Nestin, or PDGFRβ) and Cx43 with dpi (expressed as Log_10_); there was a statistically significant increase noted for GFAP and nestin with chronicity of injury. Scale bar in M is equivalent to 20 microns in A–J, 100 microns in K–N.

*Qualitative* Cx43: In the control regions (Zone 3), diffuse labelling of astrocyte‐like cells was present in the cortex (Figure S1F) and in glial cells mainly around vessels in the white matter. In acute ICR injuries, loss of Cx43 was apparent in the core of the ICR lesions, forming a sharply demarcated boundary with the adjacent parenchyma (Figures 1F, S1A); immunoreactivity at later stages was noted in relation to capillary endothelium (Figure S1B). By 8–9 dpi, Cx43 expression in the injury margin was observed in elongated cells around vessels and from subacute to intermediate lesions, Cx43 immunopositivity in the endothelium, pericyte cells and reactive multipolar cells became more evident (Figure S1C, D). In chronic lesions, the central necrotic core had contracted with intense labelling of Cx43^+^ processes demarcating the ICR scar site (Figure S1E). Aq4: In control regions (Zone 3), dense labelling of glial processes in both cortex and white matter was apparent, particularly in foot processes around blood vessels (Figure S1J). In acute ICR lesions, the loss of Aq4 immunolabelling in the injury site formed a well‐defined boundary, separating this area from the retained Aq4 immunolabelling in the adjacent viable tissue (Figure S1H), including labelling of reactive cells (Zone 1) (Figure S1I). GS: Expression was predominantly observed in the perinuclear cytoplasm of small glial cells in control regions (Figure S1M). GS^+^ cells with reactive astrocytic morphology were present in subacute and intermediate ICR lesions Zone 1 (Figure S1K) and more intensely labelled GS^+^ cells in older lesions (Figure S1L). Cx43 with nestin/GFAP: Striking membranous Cx43 expression in large, reactive nestin and GFAP positive cells was observed in acute and subacute ICR injuries in Zone 1 (Figure 5 **A,B**). In both subacute (Figure 5 **D,E**) and intermediate lesions (Figure 5 **G,H**) co‐expression of both nestin and GFAP with Cx43 was noted in a proportion of processes at the organizing margin, particularly around vessels. In chronic injuries, GFAP showed a more complete overlap with Cx43 (Figure 5 **K**) than nestin (Figure 5 **J**). In addition, large reactive Cx43^+^ cells were noted, negative for both nestin (Figure 5 **G**) and GFAP (Figure 5 **H**). Cx43 labelling of endothelium was also noted. In chronic injuries and Zone 3, the majority of GFAP reactive cells showed Cx43 labelling (Figure 5 **M**). Cx43/PDGFRβ: In acute and subacute lesions, there was very occasional co‐expression in pericytes around capillaries in the organizing margin of Zone 1 (Figure 5 **C,F**). Overall, there was less evidence for co‐expression compared to nestin and GFAP, a more distinct pattern being Cx43^+^ endothelium and surrounding PDGFRβ‐positive pericytes in organizing vessels (Figure 1 **F**, insert). Large Cx43^+^ reactive cells were typically PDGFRβ negative (Figure 5 **I**) and minimal co‐localization was observed in chronic scars (Figure 5 **L**). In Zone 3, separate distribution of PDGFRβ and Cx43 was appreciated (Figure 5 **N**); however, occasional co‐localization in small multipolar cells between vessels was observed (Figure 5 **N**, inset).

*Quantitative* Cx43: Significant differences were noted in Cx43 LI for all injuries between injury core and control region (Zone 3) (*P* < 0.005) (Table 2) with a significant linear increase in Cx43 in Zone 0 with dpi (*P* = 0.03) (Figure S1G). Aq4: There was significantly lower Aq4 LI in the lesion core (Zone 0) in all injuries compared to Zone 3 (*P* < 0.001) (Table 2) but no significant correlation of Aq4 LI with dpi. GS: There were no statistically significant differences in LI between Zones or correlation with dpi (Table 2).

*Double‐labelling quantitation:* For Cx43/nestin, there was a significant increase in the relative area of co‐localization comparing between Zones 1 and 3 (*P* = 0.038) (Table 2); this was not noted for Cx43/GFAP. There was a significant linear increase in relative area of co‐localization for both Cx43/nestin and Cx43/GFAP with dpi (*P* = 0.002 and *P* < 0.0001) (Figure 5 **O**). No statistical differences were noted for Cx43/PDGFRβ co‐localization either between Zones or with dpi (Figure 5 **O**).

## Discussion

**Figure 6:**
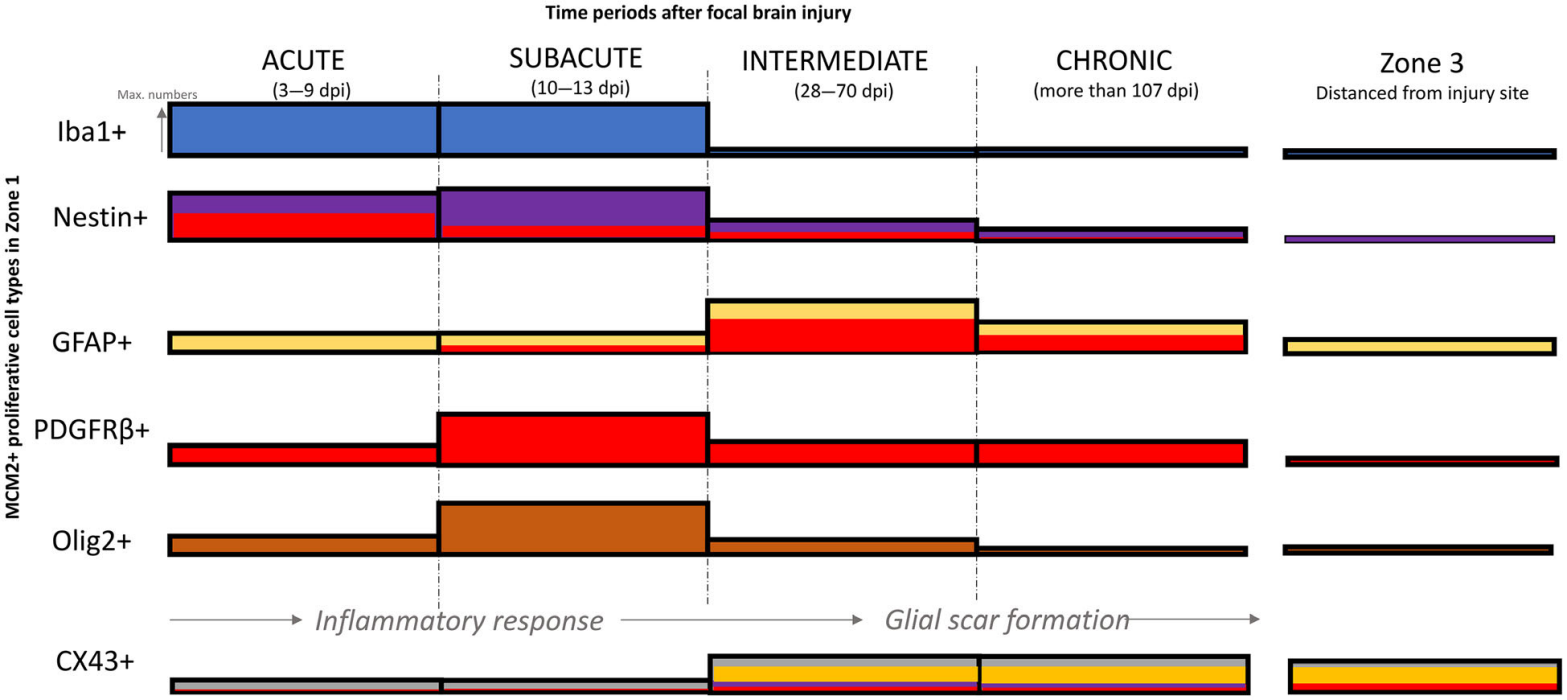
Summary schematic illustrating the expression of different proliferative cell types around the lesion at various intervals post injury. This is based on the data in current and previous study 5. The height of the bar refers to the level of expression or numbers of dividing cell types. Immediately after injury, the number of dividing Iba1^+^ microglia and nestin^+^ expressing cells are upregulated around the lesion, reaching maximal numbers around 2 weeks post lesion. At 2 weeks, an increased number of dividing PDGFRβ^+^ and Olig2^+^ expressing cells were also observed around the lesion. After a month post lesion, the number of Iba^+^ microglia and Olig2^+^ oligodendrocytes decreased dramatically, reaching similar number of cells found in Zone 3 (normal level). In contrast, many dividing GFAP ^+^ astroglia were observed only after 1 month after injury. A higher number of MCM2^+^/GFAP ^+^ cells were still found around lesion after 4 months post lesion, together with few nestin^+^ and PDGFRβ^+^ cells. The level of co‐expression between PDGFRβ^+^ and nestin (red bars within nestin^+^) was maximally observed a week after post lesion and gradually decrease with time. In contrast, the PDGFRβ^+^ and GFAP was maximally observed 2 months after lesion. The expression of CX43 was significantly reduced from normal level, immediately following injury, but increased after a month, coinciding with the upregulation of GFAP. Some PDGFRβ^+^and nestin^+^ expressing cells in Zone 1 were found to express CX43 1 month after lesion (Bar colours ‐ Blue, Iba1; purple, nestin; yellow, GFAP; red, PDGFRbeta; brown, olig2 and grey, Cx43).

Using ICR penetrating injuries in human tissues, we investigated the spatiotemporal dynamics of PDGFRβ^+^ cells and its relationship with other glial cell types such as GFAP and nestin‐expressing cells in the formation of a glial scar. Several morphological types of PDGFRβ^+^ cells were observed in lesional and perilesional tissue. PDGFRβ^+^ pericyte cells as well as Olig2^+^ glia contribute to the proliferating cell fraction in acute and subacute injury sites, in addition to the nestin^+^ cells and Iba1^+^ cells as previously described 5. PDGFRβ^+^ pericyte cells also extended away from vessels in the active organizing injuries, showed focal nestin expression, but diminished in chronic lesions. Furthermore, the relative differences and dynamic changes of Cx43 gap protein expression in glio‐vascular and reactive cell types are of potential relevance to functional changes following penetrating brain injury. Together with the data from our previous study 5 we can formulate the cellular spatiotemporal dynamics in brain repair in human tissues (Figure 6).

Needle tracks following intracranial electrode insertion provide a specific model for penetrating brain injury, without superimposed rotational or ischaemic injury and with the advantage that injuries of a precise age can be studied in optimally fixed surgical tissues. In our previous study, nestin ^+^ cells were prominent replicative cell types in ICR injuries with close relationship to vessels and remained in the chronic scar 5. In the present study, using in vitro scratch assay on primary cell cultures, proliferative nestin‐expressing cells were seen as early as 24 h after injury. Most of these cells have morphology similar to immature migrating cells. These findings are in line with observations in animal models of injury 7, 31, 32. However, the source of the nestin^+^ scar‐forming glial cells is unexplained and we speculated on their relationship to pericytes, which we further investigated in this current study.

### PDGFRβ^+^ pericyte migration and proliferation in injuries

PDGFRβ is widely recognized as a reliable marker of CNS pericytes under normal conditions 9, 13, 21. Around the ICR injury, a significant increase in PDGFRβ^+^ pericytes was observed compared to control regions, with a close spatial and temporal relationship to nestin^+^ cells, sharing similarities in cell morphology and distribution, including elongated, bipolar cell forms in proximity to new vessels. PDGFRB^+^ pericyte cells also extended away from capillary walls at the injury margin. Pericyte migration or ‘lifting’ away from micro‐vessels has been previously shown using electron microscopy in models of traumatic brain injury (TBI) at 2 days following injury 33, with loss of their contact with the blood vessels, invasion through the vascular basement membrane 34 and PDGFRβ^+^ pericyte ‘detachment’ 21. In tissues from patients with acute strokes, increased numbers of ramified pericyte cells bridging between vessels has also been reported 35. Using MCM2 co‐labelling, we also demonstrated increased replicative potential of PDGFRβ^+^ pericytes as well as in the detached cells, that peaked at 8 to 13 dpi (subacute stage). In spinal cord and brain injury models, proliferation of type 1 pericytes was shown 14 days after injury 36 and proliferation of PDGFRβ^+^ cells demonstrated with Ki67 labelling in ischaemic stroke lesions 35 as well as cortical models of TBI 37.

### Evidence for the contribution of pericytes to glial scar formation

Experimental TBI studies mainly focus on the earlier stages of repair and have demonstrated a quantitative increase in PDGFRβ^+^ cells and mRNA from 3–5 dpi 37 and 2 to 7 dpi 21. The density of PDGFRβ^+^ cells has been shown to diminish by 14 dpi 36 and by 3 months post injury, only perivascular pericytes were reported in the glial scar 21, but still with elevated numbers at 7 months compared to those before injury in another study 34. In the current study with chronic injuries of over 15 months, PDGFRβ expression was restricted mainly to perivascular cells at the scar site. In contrast, although nestin labelling overall diminished with dpi, nestin^+^ cells and processes remained, demarcating the chronic scar. A strong relationship between the LI of PDGFRβ and nestin across all stages of injuries was shown and co‐labelling identified nestin^+^/PDGFRβ^‐^, nestin^‐^/PDGFRβ^+^ and nestin^+^/PDGFRβ^+^ cells in organizing injuries. In our previous study, we reported increased co‐localization between nestin and GFAP with dpi 5 and in the current study we noted a trend for a decreased relative area of co‐localization of PDGFRβ with nestin with dpi but an increase in co‐expression with GFAP.

Although these temporal changes could reflect heterogeneous reactive cell populations during repair process (i.e. augmented nestin expression in PDGFRβ^+^ pericytes or transient PDGFRβ^+^ expression in nestin^+^ glia), an alternative explanation is that following pericyte proliferation and migration, a subset differentiates to scar‐forming glial cells 19, 34, 36. In spinal cord injury models, ablation of NG2^+^ pericyte cells reduced GFAP density in the glial scar 38. In a further study, genetic ablation of pericytes led to failure to seal the scar, implicating pericytes as a probable source of scar‐forming cells 34. Recent studies have demonstrated that the application of a PDGFR inhibitor disrupted glial scar formation following injury 39.

In experimental systems and TBI models, cellular co‐expression of PDGFRβ and GFAP in reactive cells has been shown in some 13, 19, 21, 39 but not all studies 34, 37. In a recent lineage‐tracing experiment, *Tbx*18‐expressing pericytes did not co‐localize with GFAP‐astrocytes following injury 22. In the present study, there were rare, overlapping PDGFRβ^+^/GFAP^+^ processes located close to the ICR lesions. A more striking qualitative observation in this study, however, was the ‘zonality’ or relative compartmentalization in the ICR injuries, with reactive PDGFRβ^+^ pericytes and nestin^+^ cells forming the inner border of the organizing scar with a surrounding rim of predominantly GFAP^+^ reactive glia. Such ‘layering’ of organizing scars has been previously noted in experimental models 34, 38 as well as in human strokes where PDGFRβ^+^ proliferating cells were demarcated from the surrounding astrocytic gliosis 35. The findings of the present study therefore support two components to brain repair: the formation of a central, chronic, contracted glial scar, dependent on early proliferation and then decline of PDGFRβ^+^ and nestin^+^ cell types and an outer predominantly GFAP^+^ glial scar.

### PDGFRβ^+^ CNS parenchymal cells

There is accumulating evidence that PDGFRβ is also expressed in resting brain parenchymal cells other than pericytes. We previously reported, in the white matter of adult epilepsy cases with FCD, small multipolar cells, away from capillaries, which co‐expressed PDGFRβ and PDGFRα as well as NG2 and likely represented subsets of NG2 progenitor glia 40. An increase in ramified PDGFRβ^+^ parenchymal cells was observed in human temporal lobe epilepsy (TLE) that co‐localized with NG2 but not Iba1 41 and their redistribution showing the following experimental status suggests that they are reactive populations 42. Kyyriainen *et al*., recently described PDGFRβ^+^ cells with small soma and ramified processes in normal mice brain shown to be either PDGFRα^+^ or GFAP^+^ and dynamic changes in their distribution was also shown following both experimental status and trauma 21. In the present study small PDGFRβ^+^ parenchymal cells were more evident away from the injury site in normal white matter; they showed focal co‐expression with nestin (and to lesser extent GFAP) but not for SMA, unlike pericytes. We cannot exclude that such pre‐existing parenchymal PDGFRβ^+^ glia also contribute to reactive, proliferating populations at injury sites.

Oligodendrocyte lineage progenitors (OPCs) are in close apposition with pericytes in the perivascular space, with evidence for mutual interactions, promoting proliferation 43. Surgical TBI resections have shown an increase in OPC number at injury site 44 and transient proliferation of OPC noted between 7 to 21 dpi in a diffuse TBI model 45. In our study significant proliferation was confirmed, with Olig2/MCM2^+^ cells peaking at 13 dpi then falling to control levels in chronic lesions. The morphology of Olig2/MCM2^+^ remained ‘oligo‐like’ with no definite evidence of glial/pericyte maturation; however, no double labelling of Olig2 with nestin or PDGFRβ was performed in the present study. We have also recently identified nestin^+^ radial glial cell types in adult temporal lobe with regenerative capacity, glial maturation *in vitro* and focal co‐localization with PDGFRβ 8. Heterogeneous populations of regenerative parenchymal cells could therefore potentially contribute to astroglial scar formation following injury. Further work is needed to define their cellular interactions and signalling pathways that are pivotal to optimal brain repair.

### Connexin43 and functional glial markers

Expression of astroglial markers Cx43, Aq4 and GS in reactive cell types is a further evidence of their differentiation and also reflects functional alterations over the time course of brain repair, of potential relevance to epileptogenesis and other post‐traumatic sequela 23. Cx43 is the main gap junction protein in astrocytes 24 and Cx43 hemichannels, mediating ‘gliotransmission’ and release of bioactive molecules from astrocytes 25, 26. Both connexins and Aq4 are implicated in spreading depolarization following injury 27, 28. Connexins are also critical to cell‐cell interactions and establishing homeostatic cell communication following injury 46, driving cell reactivity 3 and promoting neuronal recovery 47; for example, the extent of gliosis is larger in Cx43 KO mice 48.

We noted spatiotemporal alterations in expression, with initial reduction at the injury site for Cx43 and Aq4 labelling compared to control regions but increased labelling of Cx43 with dpi and its cellular co‐expression with GFAP (see summary Figure 6). In experimental TBI models, an initial decrease in Cx43 was also reported followed by an increase from 6 to 15 dpi 48. We noted only occasional co‐localization of Cx43 with perivascular PDGFRβ^+^ pericytes but exaggerated endothelial labelling; loss of Cx43 in pericytes has been linked to functional decline and vascular instability 49 and increased endothelial expression with defective blood‐brain barrier 50. Dynamic changes in Cx43 expression in the neurovascular unit in brain repair warrant further investigation regarding physiological versus detrimental role.

### Limitations

All of the patients had refractory focal epilepsy and it is likely that seizures influence the baseline glial populations, including densities of nestin 8, PDGFRβ^+^ glial cells 41, 42 and Cx43 expression on astrocytes 24 in Zone 3 used as the control region as in previous studies. Nevertheless, we favoured this as optimal control tissue over post mortem tissue with agonal changes and delayed and longer fixation times which could affect staining.

In summary, in the ICR brain injury model we have identified that (i) PDGFRβ^+^ and Olig2^+^ cells contribute to the proliferative fraction with evidence of pericyte migration away from the vessels into the organizing scar, (ii) the formation of a glial scar is composed of two Zones, the central Zone enriched in nestin^+^ pericytes and an outer Zone of reactive GFAP‐astrocytes, (iii) PDGFRβ also identifies populations of small neuroglial cells and (iv) glial differential markers, as Cx43, show altered distribution in reactive cells and the neurovascular unit during repair which could have functional implications.

## Author contributions

JL, CR and APJ were involved in the section preparation and analysis. MT was involved in data collection and study design. JL and MT conducted all the data analysis and interpretation. JL, CR and MT were involved in manuscript preparation. SS conceived the initial injury study design and reviewed the manuscript.

## Supporting information


**Figure S1.** Functional astrocytic markers Cx43, Aq4 and GS in ICR injuries.

**Figure S2.** Immunofluorescence images shown as split channels in green and purple.

**Figure S3.** Nestin, PDGFRβ and GFAP labelling with injury age.

**Table S1.** Markers for chromogenic and immunofluorescence studies.

**Method S1.** Supplementary Methods.
