# Supplementary figures and images for: Spatiotemporal dynamics of PDGFRβ expression in pericytes and glial scar formation in penetrating brain injuries in adults
Source: Neuropathol Appl Neurobiol. 2019 Apr 2;45(6):609–27. doi: 10.1111/nan.12539 (PMC6767497; doi:10.1111/nan.12539)

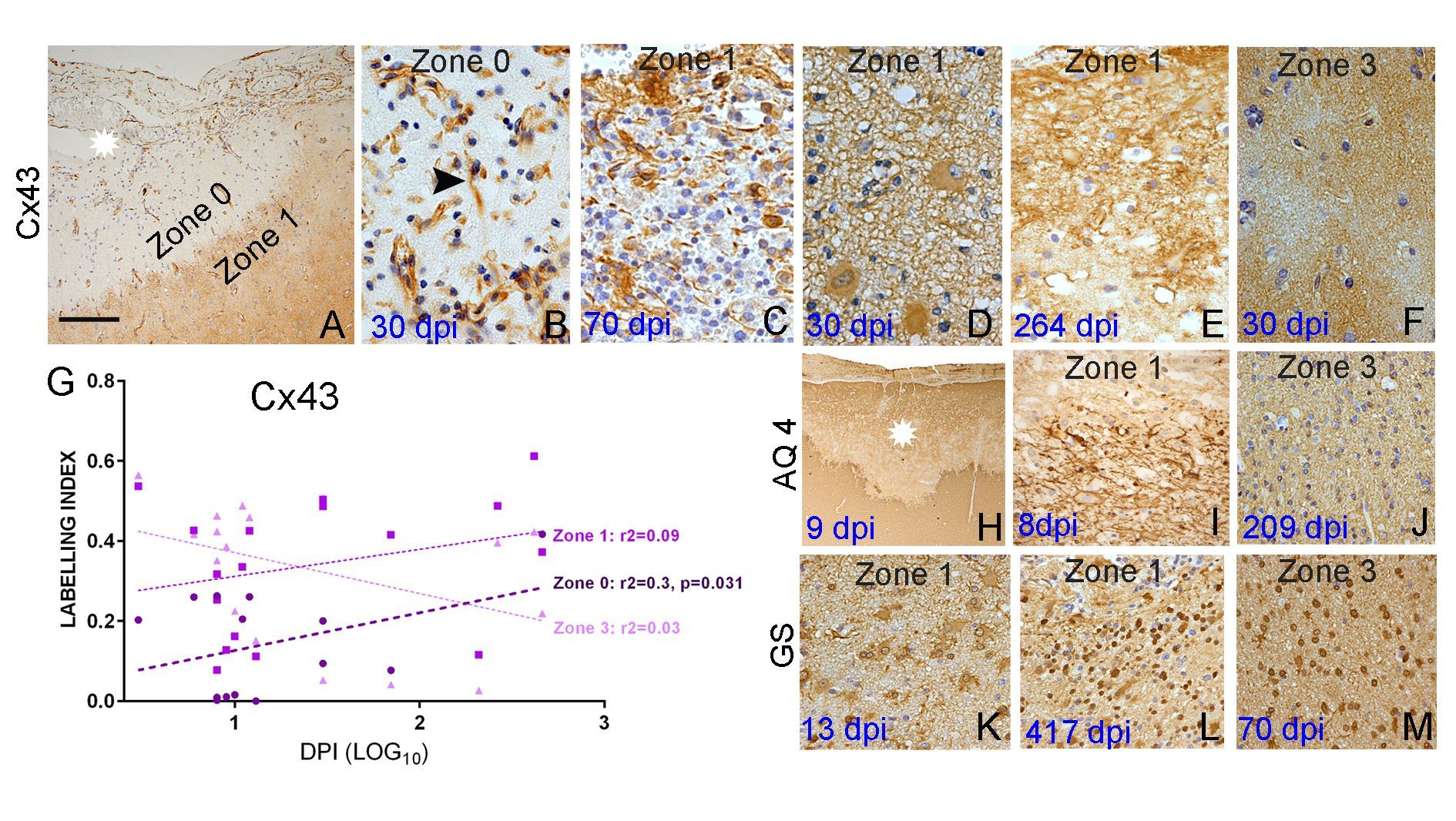

Supplement: Supplementary file 1 — Figure S1. Functional astrocytic markers Cx43, Aq4 and GS in ICR injuries. [file NAN-45-609-s001.jpg]

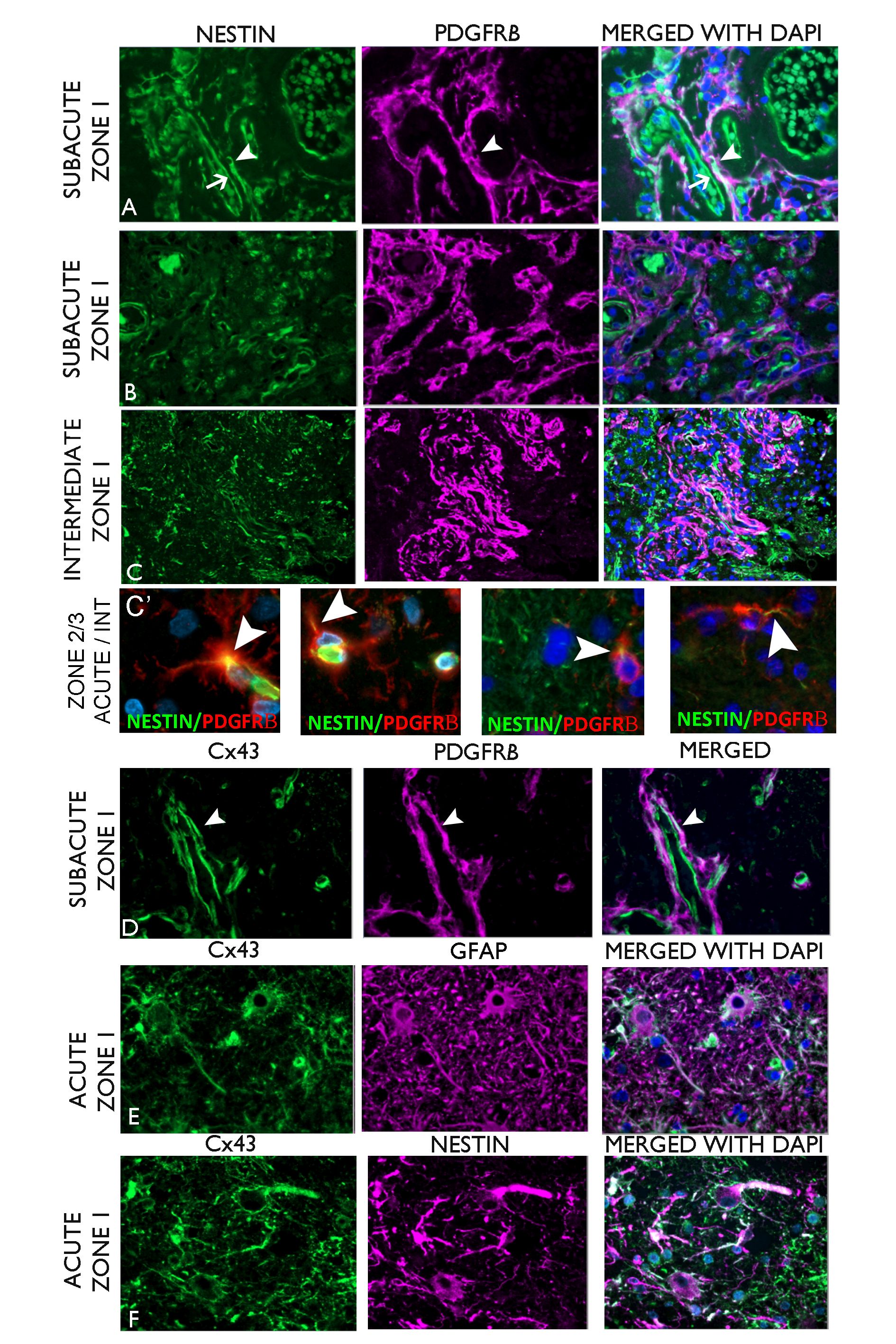

Supplement: Supplementary file 2 — Figure S2. Immunofluorescence images shown as split channels in green and purple. [file NAN-45-609-s002.jpg]

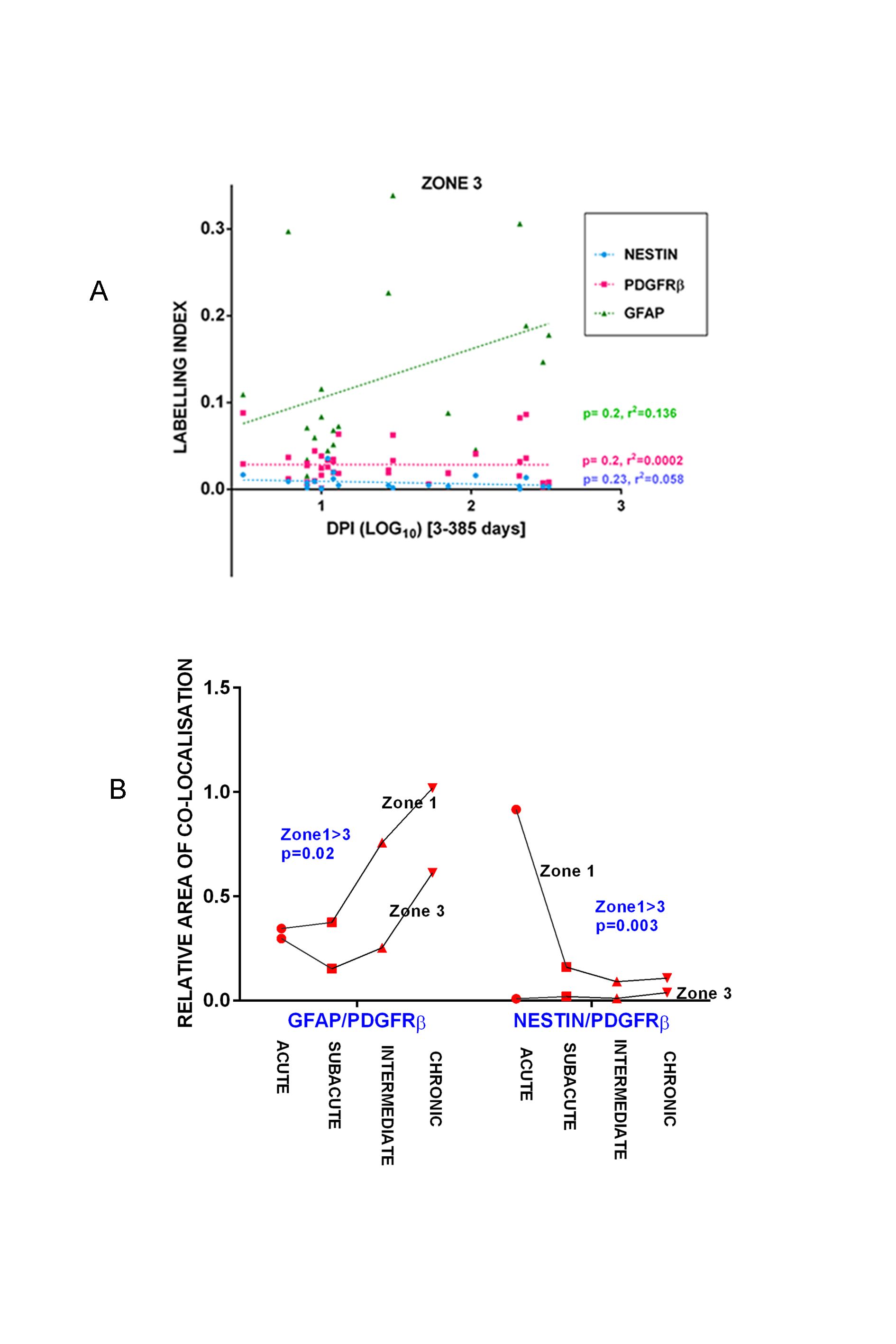

Supplement: Supplementary file 3 — Figure S3. Nestin, PDGFRβ and GFAP labelling with injury age. [file NAN-45-609-s003.jpg]
