# Supplementary material for: Spatiotemporal dynamics of PDGFRβ expression in pericytes and glial scar formation in penetrating brain injuries in adults
Source: Neuropathol Appl Neurobiol. 2019 Apr 2;45(6):609–27. doi: 10.1111/nan.12539 (PMC6767497; doi:10.1111/nan.12539)
Supplement: Supplementary file 4 — Table S1. Markers for chromogenic and immunofluorescence studies. [file NAN-45-609-s004.docx]

| **Antibodies**  Clone, Code | **Immunogen or target epitope** | **Labelled cell or protein type in the brain** | **Antibody supplier,** antibodies dilution, incubation conditions |
| --- | --- | --- | --- |
| **Anti-Nestin**  10C2, AB22035 | 150 aa recombinant fragment from human nestin conjugated to GST | Immature progenitors, glia, endothelial cells | EMD; 1:1000, ov |
| **Anti-PDGFRβ**  Y92, AB32570 | Synthetic peptide within human PDGFRβ aa 1050 to the C-terminus | Marker for Pericytes and some NG2 cells | Abcam Plc.; 1:1000, ov |
| **Anti-GFAP**  Z0334 | GFAP isolated from cow spinal cord | Intermediate filament in mature Astrocytic cells | DAKO; 1:1500, ov |
| **Anti-SMA**  MO851 | N-terminal synthetic decapeptide of alpha-smooth muscle actin | Smooth muscle cells, myofibroblasts and myoepithelial cells | DAKO: 1:500 ov |
| **Anti-Aq4**  A5971 | Recombinant fusion protein to residues 249-323 of rat AQP4 fused to GST | Aquaporin 4  Functional marker for mature astrocytes | Sigma; 1:500; ov |
| **Anti-Cx43**  CX-1B1, 13-8300 | Synthetic peptide to a cytoplasmic sequence located near the C-terminus of rat Cx43 | Connexin 43  Gap junction/hemi-channel Marker for Astrocytes | Thermo Fisher; 1:500; ov |
| **Anti-GS**  GS-6, MAB302 | purified from sheep brain. | Glutamine synthetase Marker for functional Astrocytes | EMD; 1:500; ov |
| **Anti-MCM2**  46/BM28, 610700 | Human BM28 aa. 725-888 | Mini-chromosome maintenance protein 2  Cells licensed for replication | BD; 1:900; ov |
| **Anti-Olig2**  AB9610 | Recombinant mouse Olig-2 | Nuclear based basic helix-loop-helix (bHLH) transcription factor Oligodendrocyte lineage, NG2-expressing glial progenitors,  postmitotic oligodendrocytes | EMD; 1:250; ov |

Supplementary table: Markers for chromogenic and immunofluorescence studies. All sections were pre-treated in antigen retrieval buffer (H-3301, Vector Labs, UK), except for anti-Olig2, which require pre-treatment in sodium citrate buffer, pH6, prior to primary antibody incubations. Cx43= connexin 43, GFAP, glial fibrillary acidic protein; GS, glutamine synthetase; ov, overnight at 4°C; MCM2, mini chromosomal maintenance 2; PDGFRβ, platelet derived growth factors receptor beta. Suppliers: Abcam plc., Cambridge, UK; BD Transduction Lab., Oxford, UK; DAKO, Cambridgeshire, UK; EMD Millipore, Watford, UK; Sigma Aldrich Company Ltd, Dorset, UK; Thermo Fisher Scientific, Hemel Hempstead, UK.
