# Supplementary material for: Spatiotemporal dynamics of PDGFRβ expression in pericytes and glial scar formation in penetrating brain injuries in adults
Source: Neuropathol Appl Neurobiol. 2019 Apr 2;45(6):609–27. doi: 10.1111/nan.12539 (PMC6767497; doi:10.1111/nan.12539)
Supplement: Supplementary file 5 — Method S1. Supplementary Methods. [file NAN-45-609-s005.docx]

Supplementary Methods

**Single labelling immunohistochemistry (CX43, Aq4, GS)**

Fixed brain sections were deparaffinized in xylene and rehydrated in alcohol. Endogenous peroxidase was blocked by immersing the sections in 0.9% hydrogen peroxide for 15 minutes. The sections were then microwaved at 800W for 12 minutes in antigen retrieval buffer (H3301; Vector Laboratories Ltd., Peterborough, UK) and allowed to cool for 20 minutes. Blocking solution consisting of 2.5% normal horse serum (Vector Laboratories, Peterborough, UK) was applied for 20 minutes. Sections were then incubated overnight at 4°C with the first set of diluted primary antibodies (Table 2). On the following day, species specific HRP-conjugated secondary antibodies were applied for 30 mins before chromogenic detection. Immunolabelled sections were counterstained in haematoxylin and processed through ascending concentrations of alcohol and xylene before coverslipped.

**Double labelling methods**

**(Nestin/PDGFRβ, PDGFRβ/GFAP, CX43/PDGFRβ, CX43/GFAP and CX43/Nestin, olig2/MCM2, PDGFRβ/SMA)**

Immunolabelled sections were underwent pre-treatment and blocking procedures as described above. Primary antibodies solution consisted of the following markers (Table 2): MCM2, PDGFRβ, GFAP, nestin, CX43, and/or olig2. On the following day, species specific HRP-conjugated secondary antibodies were applied for 30 minutes at room temperature, and fluorescein-labelled antibody in tyramide signal amplification buffer (TSA; Perkin Elmer, Buckinghamshire, UK) was applied for eight minutes. Following a ten minute incubation in 0.9% hydrogen peroxidase, the second set of primary antibodies was added overnight at 4^o^C. On the following day, species specific HRP-conjugated antibodies were applied for 30 minutes at room temperature, and then Cy3-labelled antibodies in TSA buffer was applied for eight minutes. Sections were coverslipped using Vectashield mounting media with DAPI (Vector Laboratories Ltd., Peterborough, UK). In negative controls, one or both primary antibodies were omitted.

Chromogenic double labelling was performed using markers, PDGFRβ and MCM2. The protocol for double labelling was similar as described above except that DAB and VIP were used instead of fluorescein and Cy3-conjugated antibodies. Labelled sections were counterstained in haematoxylin and processed through ascending concentrations of alcohol and xylene before coverslipped.

***In vitro* scratch assay**

Cells were cultured from a gram of fresh tissue sampled from the grey and white matter of the temporal pole of two surgical patients with MTLE and ILAE Type 1 HS (Cases EC1-2). Dissociated cells were cultured in supplemented MACS Neuro Medium (30,000 cells/mL) for four weeks using a protocol established in our previous study [1]. Prior to the mechanical scratch assay, a confluent monolayer of cells was grown on Fluorodish (World Precision Instruments, Florida, USA). A vertical scratch was made in the middle of the culture using a 100 µm pipette tip. 10µM of 5-ethynyl-2’-deoxyuridine (EdU A; Thermo Fisher Scientific, Massachusetts, USA), a modified thymidine analogue [2] was added to the culture medium 24 hours after mechanical injury, for thirty minutes at 37^o^C, to identify cells that were actively undergoing DNA synthesis. After incubation with EdU A solution, cells were fixed using 4% paraformaldehyde (Santa Cruz Biotechnology, Heidelberg, Germany), and double labelled immunocytochemistry was performed using reagents supplied in Click-iT EdU Imaging kit (Thermo Fisher Scientific, USA), and primary antibodies against nestin. First, EdU A was labelled with Alexa-564 conjugated antibodies following manufacture’s instruction (Thermo Fisher Scientific, UK). Anti-nestin antibody diluted in PBS with 10% normal horse serum was applied at 1:1000 overnight at 4ºC. The following day, species-specific Alexa-488 conjugated secondary antibodies were applied for ninety minutes at room temperature, and Hoechst solution was applied for thirty minutes. Labelled preparations were visualised, and images were acquired using confocal microscopy (LSM700; Zeiss, Germany). Five serial images spanning 7000 µm along the horizontal axis were acquired at three points along the vertical axis of the scratch (top, middle, bottom). Images were imported into the image analysis software, Definiens Tissue Studio 3.6 and Developer X64 (Definiens AG; Munich, Germany) for automated quantification. Final results were expressed as the percentage of cells that have incorporated EdU A and expressed nestin at various distance along the x-axis from the scratch border. Statistical analysis was not undertaken due to the limited number of samples available for *in vitro* studies.

1 Liu J, Reeves C, Jacques T, McEvoy A, Miserocchi A, Thompson P, Sisodiya S, Thom M. Nestin-expressing cell types in the temporal lobe and hippocampus: Morphology, differentiation, and proliferative capacity. Glia 2018; 66: 62-77

2 Nakayama D, Matsuyama T, Ishibashi-Ueda H, Nakagomi T, Kasahara Y, Hirose H, Kikuchi-Taura A, Stern DM, Mori H, Taguchi A. Injury-induced neural stem/progenitor cells in post-stroke human cerebral cortex. The European journal of neuroscience 2010; 31: 90-8
